# Between simplicity and accuracy: Effect of adding modeling details on quarter vehicle model accuracy

**DOI:** 10.1371/journal.pone.0179485

**Published:** 2017-06-15

**Authors:** Ming Foong Soong, Rahizar Ramli, Ahmad Saifizul

**Affiliations:** Advanced Computational and Applied Mechanics (ACAM) Research Group, Department of Mechanical Engineering, Faculty of Engineering, University of Malaya, Kuala Lumpur, Malaysia; Chongqing University, CHINA

## Abstract

Quarter vehicle model is the simplest representation of a vehicle that belongs to lumped-mass vehicle models. It is widely used in vehicle and suspension analyses, particularly those related to ride dynamics. However, as much as its common adoption, it is also commonly accepted without quantification that this model is not as accurate as many higher-degree-of-freedom models due to its simplicity and limited degrees of freedom. This study investigates the trade-off between simplicity and accuracy within the context of quarter vehicle model by determining the effect of adding various modeling details on model accuracy. In the study, road input detail, tire detail, suspension stiffness detail and suspension damping detail were factored in, and several enhanced models were compared to the base model to assess the significance of these details. The results clearly indicated that these details do have effect on simulated vehicle response, but to various extents. In particular, road input detail and suspension damping detail have the most significance and are worth being added to quarter vehicle model, as the inclusion of these details changed the response quite fundamentally. Overall, when it comes to lumped-mass vehicle modeling, it is reasonable to say that model accuracy depends not just on the number of degrees of freedom employed, but also on the contributions from various modeling details.

## Introduction

It is probably known that virtual development has become an integral part in vehicle development process due to the shortening of product life cycle in the automotive industry. Be it in the development of conventional vehicles or even in the research of state-of-the-art vehicle technologies like electric vehicle and hybrid electric vehicle [1–3], virtual development through simulation is both time and cost effective compared to physical prototype testing as it allows fine-tuning and optimization of a vehicle to be performed efficiently. For example, an important phase of virtual vehicle development is the simulation of vehicle dynamics to achieve optimum ride and handling performances. Obviously, such simulation requires an accurate representation of the vehicle, often called vehicle model, for meaningful result analysis and interpretation.

There are a number of vehicle models that are meant for vehicle dynamic simulation [4–6], covering a range of model complexities and degrees of freedom (DOFs). However, it is safe to say that all these originate from two major classifications based on modeling approach. Generally, there are two types of vehicle modeling: the first type considers a vehicle to consist of detailed individual rigid bodies that connect with one another kinematically or dynamically to form a detailed assembly equivalent to the entire vehicle system. Such models are called multi-body models. Meanwhile, the second type of modeling considers various parts of the vehicle as lumped masses and thus considers only the essential DOFs associated with the few lumped masses, such as vehicle body and wheels. These models are logically named lumped-mass vehicle models. Conventionally, multi-body modeling is seen to be capable of predicting the actual vehicle response accurately. In fact, many have employed multi-body models in their studies [7–9] and have demonstrated good correlation between simulated and actual responses during model validation. On the contrary, lumped-mass vehicle models are typically regarded as being less accurate than multi-body models. To give an example, Na and Yoo [10] argued that lumped-mass models are not adequate for dynamic analysis as the various vehicle sub-systems are regarded as lumped rigid parts for model simplification, and this does not accurately express the forces acting on vehicle body and wheels.

Of the many lumped-mass vehicle models, quarter vehicle model is the most basic representation of a vehicle as it consists of only two DOFs, namely sprung and unsprung mass motions. However, the scenario for quarter vehicle model is an interesting one: on one hand, this model is widely used in researches involving vehicle and suspension analyses, particularly those related to vertical vehicle dynamics or ride dynamics. This is mainly due to model simplicity [11]. On the other hand, its accuracy, or rather the lack of it, is also brought up as a major opposing view, also because of the same simplification in modeling where many features are not represented due to the limited DOFs. In fact, this concern on accuracy is often accepted, often without further investigation or quantification. So this interesting situation brings up a few questions worth pondering: (i) what is the limit of a quarter vehicle model? Specifically, can the accuracy of this model be stretched while retaining the same DOFs, for instance through refinement in modeling details? Extending from this, a more important question is: (ii) what is the significance of these modeling details? Are they worth the inclusion in, or exclusion from, a quarter vehicle model? Considering the vast adoption of quarter vehicle model in vehicle dynamic study, these are definitely worth answering.

This study provides some clarifications by focusing on the effect of adding various non-DOF-related modeling details on the vehicle response. In the study, four modeling details applicable to quarter vehicle model, namely road input detail, tire detail, suspension stiffness detail and suspension damping detail were identified and investigated. These acted as refinements to the otherwise linear and basic quarter vehicle model, and several enhanced models were compared to the base model through simulated responses. It was found that certain modeling details, like the road input detail and the suspension damping detail, are worth adding for improved accuracy, while others are not. These will be elaborated in the sections that follow.

## An insight into quarter vehicle model

**Fig 1:**
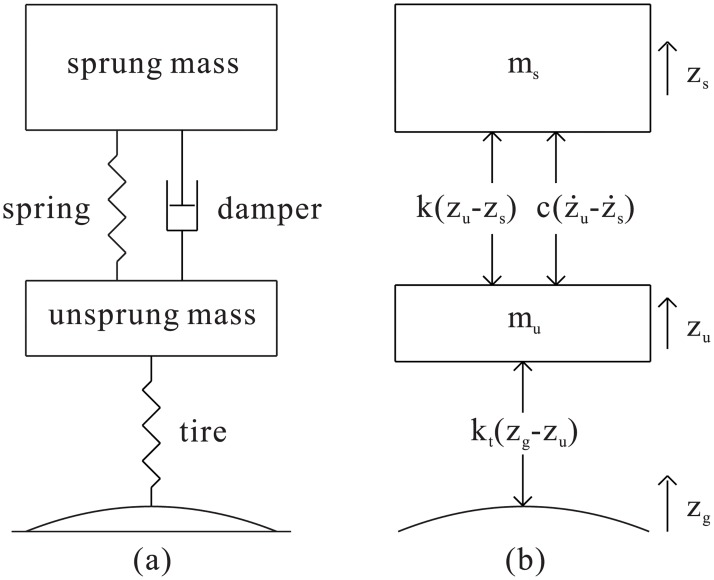
(a) Schematic representation of a lumped-mass quarter vehicle model and (b) its free-body diagram.

Quarter vehicle model is the simplest representation of a vehicle in dynamic analysis. It consists of the most essential DOFs that describe the movement of a vehicle, namely the sprung mass motion and the unsprung mass motion, all in the vertical translational direction. The lumped-mass approach in quarter vehicle modeling implies that there are only two inertial components which are the sprung mass and the unsprung mass, consistent to the two DOFs stated earlier. For a quarter vehicle model, the sprung mass represents loosely the quarter mass of vehicle body which includes all parts supported by the suspension system. Meanwhile, the unsprung mass comprises the masses of all parts of a single wheel station that are acted by the suspension. Due to the two DOFs, a typical lumped-mass quarter vehicle model is basically capable of representing the body bounce and wheel hop modes of movement of a vehicle. Mathematically, these motions can be described by the two equations of motion (Eqs (1) and (2)) which can be derived from the schematic model shown in Fig 1.

$$m_{s}{\ddot{z}}_{s}=k\left(z_{u}-z_{s}\right)+c\left({\dot{z}}_{u}-{\dot{z}}_{s}\right)$$ (1)

$$m_{u}{\ddot{z}}_{u}=k_{t}\left(z_{g}-z_{u}\right)-k\left(z_{u}-z_{s}\right)-c\left({\dot{z}}_{u}-{\dot{z}}_{s}\right)$$ (2)

in which *m*_*s*_, *m*_*u*_ are the sprung and unsprung mass values, *z*_*s*_, *z*_*u*_ are the vertical sprung and unsprung mass displacements that represent the DOFs, their derivatives are the corresponding velocities and accelerations, *z*_*g*_ is the vertical road input displacement, while *k*, *c*, *k*_*t*_ are the dynamical representations of the system, namely suspension stiffness, suspension damping and vertical tire stiffness. The dynamical components of a quarter vehicle model are typically simplified, or linearized in this context: suspension stiffness is usually represented by linear or constant spring rate, suspension damping is usually treated as being linearly proportional to velocity, and the tire representation is usually assumed to have point contact with linear vertical stiffness but negligible damping. In general, this simplistic, lumped-mass, linear quarter vehicle model is the variant that is most widely used in vehicle-related studies.

The two-DOF quarter vehicle model has numerous applications. It is mostly used to study vertical vehicle dynamics, especially due to ground excitation which is the road input. This allows the common performance parameters, such as the root-mean-squared (RMS) sprung mass acceleration, dynamic tire load and suspension travel to be obtained for ride performance evaluation. Considering this, and also due to its fundamental nature, quarter vehicle model often finds its place in studies relevant to fundamental suspension concepts, such as controllable suspensions and a recently introduced new suspension element known as inerter. In fact, many well-known semi-active suspension control strategies, including Skyhook [12], Groundhook and hybrid strategies [13] were proposed and investigated through this model. It follows that many relevant comparative studies also employed the same model [14]. Similarly, the ride performance benefit of vehicle suspensions with inerter has consistently been proven in various studies [15–19], all through analysis using quarter vehicle model. In addition, quarter vehicle model has also been employed in vehicle or suspension optimization work as well [20, 21], although the involvement of lumped-mass vehicle models are sometimes limited to initial or first optimization only. Supportive to this view, Tey et al. [22], and Tey and Ramli [23] showed that lumped-mass vehicle models are still useful in early suspension optimization as a predictive model to locate a region of interest for the parameters to be optimized before using multi-body models for detailed fine-tuning.

Observing from all the applications above, it appears that the fundamental nature and the implied modeling convenience are the only reasons for the adoption of quarter vehicle model, but in so doing, the issue of modeling accuracy has to be tolerated or dealt with through control methods that are robust to uncertainties. This is the description of current scenario. The truth is, the use of quarter vehicle model in vehicle analyses has always received mixed views. The major supportive view is that this model is used due to model simplicity [11, 20]. According to Georgiou et al. [20], it is capable of providing qualitatively correct information, especially for ride studies that involve low forcing frequencies. However, on the opposition side, a quarter vehicle model does not contain detailed representations of a vehicle, making the prediction of realistic vehicle response challenging. This gives rise to the concern of model accuracy. To elaborate further, ElMadany and Abduljabbar [24] mentioned that when detailed vehicle motion is required, more elaborate models which take into account of features omitted from this model must be used.

While the lack of accuracy for quarter vehicle model is probably true, this concern is unfortunately often accepted without further explorations. Lumped-mass vehicle models with greater DOFs usually receive decent attention in terms of model accuracy, as demonstrated by various dedicated validation studies [4–6]. Ironically, such attention is not present for the elementary quarter vehicle model despite its wide adoption. The closest to this is perhaps the study by Maher and Young [25], in which comparison was done among a linear quarter vehicle model, its corresponding non-linear model and an actual quarter vehicle rig, subjected to narrowband excitation. It was found that a non-linear model produced results that were significantly more accurate than linear model. However, it is observed that the study focused mostly only on the suspension damping characteristic, and inferences on accuracy were based on quarter vehicle rig equipped with physical components. Meanwhile, the current study has a different approach by evaluating the effect of multiple available modeling details for quarter vehicle model on the predicted vehicle response, considering the context of model simulation. This gives an understanding on quarter vehicle model accuracy from a different perspective.

## Method of study

In this study, vehicle modeling details are taken to be the test subjects. Although the typical lumped-mass quarter vehicle model is highly simplified to the point that some features simply cannot be incorporated to the model, there are still various areas that can be enhanced by the corresponding modeling details. Referring back to the schematic model in Fig 1, it is immediately recognizable that a quarter vehicle model consists of only a few components, namely the sprung mass, unsprung mass, suspension stiffness, suspension damping, tire representation and, finally, the road displacement input which forms the entire system together with the other model components. For the two inertial components, there is basically no possibility of enhancement without moving to higher-DOF models. Excluding the inertial components, the remaining four components of a quarter vehicle model can all be subjected to enhancements by the incorporation of modeling details. These corresponding details, named in this study as road input detail, tire detail, suspension stiffness detail and suspension damping detail, serve as the subjects for evaluation and are described here.

**Fig 2:**
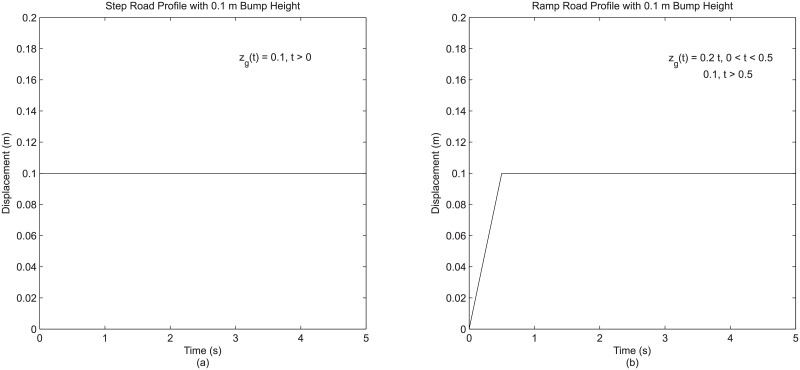
(a) Step road profile and (b) ramp road profile used in this study.

The first description is related to road input. Among the four modeling details stated above, road input is the only detail that is external to quarter vehicle model. Strictly speaking, road input is not part of the model, but it is definitely part of the entire two-DOF system that is to be simulated. In fact, it is commonly known that there are three elements associated with a typical vehicle dynamic simulation, with vehicle and road being two of them (the other is the driving maneuver which is not relevant here as quarter vehicle model is meant only for ride or vertical vehicle dynamics). Road input is still included as one of the details, as it is the only direct input to a quarter vehicle system and is logically capable of affecting the output or vehicle response. For this study, a transient input emulating the scenario of a vehicle being driven across a bump was chosen as the road input. The most elementary but commonly used representation of this scenario is the step profile which was used in the base quarter vehicle model for reference. A possible enhancement can be achieved through the use of ramp profile as the road input detail. This detail is a much more realistic representation of a bump as it is highly reminiscent of an actual trapezoidal-shaped bump. For further illustration, the exact mathematical representation of both road profiles used in this study are shown in Fig 2.

Meanwhile, for tire component, the usual modeling approach for a typical linear quarter vehicle model is to represent the vertical tire property by linear tire stiffness, assuming constant point contact with the road and also without tire damping. This formed the basis of tire modeling for the base model in this study for comparison with the enhancement. The tire detail is in the form of the addition of linear tire damping alongside the existing tire stiffness, similarly with constant point contact assumption. Actually, more involved vertical tire modeling using non-linear tire properties can possibly be added as part of the detail; however, the presence of tire damping in a quarter vehicle model is put into focus here as the expectation is that tire damping should have direct influence on vehicle response, particularly the unsprung mass response. In fact, tire damping is a subject that has received mixed views, with study that goes for its inclusion in vehicle modeling and study that mentions its insignificance [25]. Considering its vague status in vehicle modeling, it is interesting to treat this as the detail for investigation.

**Fig 3:**
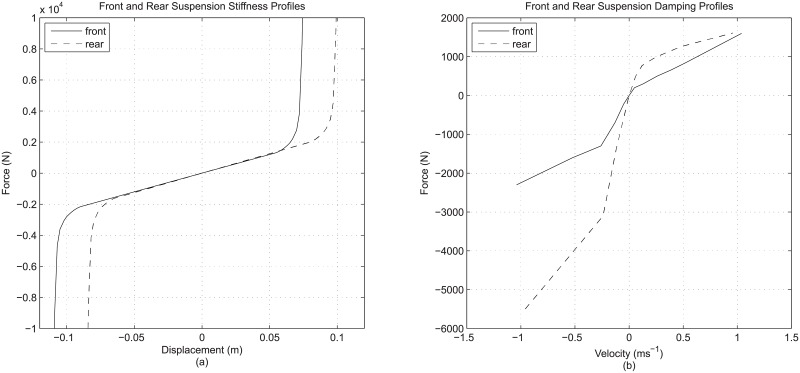
Front and rear profiles for (a) suspension stiffness and (b) suspension damping.

Finally, for suspension modeling of a quarter vehicle model, the suspension details consist of these two: the stiffness detail and the damping detail. These are seen as two separate details in this study. For suspension stiffness, the base model employed the common approach of using linear or constant stiffness value as the representation, while the enhanced model adopted stiffness detail in the form of non-linearity of the overall suspension stiffness contributed by the inclusion of suspension bushing property. In reality, the property of a physical suspension spring is generally linear, and the basic modeling approach of using linear stiffness value (constant spring rate) is probably sufficiently close. Consequently, instead of focusing on the rather insignificant non-linearity of the suspension spring, the overall non-linear stiffness due to inclusion of bump-stop and rebound-stop definitions was taken as the suspension stiffness detail. For clarity, the exact stiffness profiles adopted in the study are shown in Fig 3a. In contrast, for suspension damping, a physical viscous damper used in vehicle suspension contains inherent non-linear property that is very different from the linear damping approximation used in a typical quarter vehicle model. Therefore, while the base model employed linearized damping for reference purpose, the enhanced model considered damping detail incorporating non-linear damping property which was defined numerically using lookup table representation with linear interpolation and extrapolation. Similar to suspension stiffness, the exact damping profiles adopted in the study are shown in Fig 3b.

With four modeling details in mind, the effect as well as the significance of these details on vehicle response can primarily be evaluated in the study by comparing between the base quarter vehicle model and several enhanced models with the addition of each detail individually. However, to ensure comprehensiveness, the study considered all possible cases involving single and combined addition of details. Following basic factorial design, with four factors to be investigated (modeling details) and each consisting of two states (with or without the inclusion of detail), a total of 16 combinations were possible for analysis. These possible cases, together with the description of the states of various modeling details, are summarized and stated in Table 1.

**Table 1:** Test cases and the corresponding states of modeling details.

Test case	Road input detail	Tire detail	Suspension stiffness detail	Suspension damping detail
*No detail added*				
1	0	0	0	0
*One detail added*				
2	1	0	0	0
3	0	1	0	0
4	0	0	1	0
5	0	0	0	1
*Two details added*				
6	1	1	0	0
7	1	0	1	0
8	1	0	0	1
9	0	1	1	0
10	0	1	0	1
11	0	0	1	1
*All but one details added*				
12	1	1	1	0
13	1	1	0	1
14	1	0	1	1
15	0	1	1	1
*All details added*				
16	1	1	1	1

Note:

0 = without detail, 1 = with detail

Road input detail: 0 = step profile, 1 = ramp profile

Tire detail: 0 = only tire stiffness, 1 = with tire damping

Suspension stiffness detail: 0 = linear stiffness, 1 = non-linear stiffness with stops

Suspension damping detail: 0 = linear damping, 1 = non-linear damping

As observed from Table 1, case 1 represents the base model with the exclusion of all the stated modeling details and serves as the starting point for detail addition, while cases 2 to 5 represent the four different enhanced models due to the inclusion of each modeling detail individually. With case 1 as the reference, the vehicle responses for cases 2 to 5 basically allow for determination of the effect of each detail. Meanwhile, cases 6 to 11 represent the combined and simultaneous addition of modeling details, and cases 12 to 15 can be interpreted reversely as having the omission of each detail from the fully enhanced model (case 16) in which all four details were present. Taking case 16 as the reference, by reverse deduction, cases 12 to 15 allow for determination of the accuracy contribution and the significance of each detail, with the core assumption in this study being that the closer a mathematical vehicle model is to the physical representation, the more accurate the predicted response will be.

In the study, the vehicle parameter values were extracted from the specifications of a generic vehicle available from a commercial vehicle simulation software. The vehicle data set refers to a general mid-sized passenger vehicle which can be treated as the typical vehicle representation in most situations. The parameter values relevant to quarter vehicle model are listed in Table 2. Some of these, such as the suspension stiffnesses and linear tire properties, were adopted directly from the vehicle data set. Conversely, some other parameter values had to be derived from the source data set. For example, the front and rear sprung mass values for use in quarter vehicle model were reduced from the full vehicle body, considering left-right symmetry and the equivalent moment about the vehicle’s center of mass. Meanwhile, the front and rear damping rates in Table 2 were the result of linearization from the original non-linear damping profiles as in Fig 3b. For completeness of the result analysis, both front and rear quarter vehicle parameter values were adopted in the various cases described in Table 1. It should be noted that each of the cases is a complete and standalone simulation system as it already comprises the model and the input. Therefore, all the 16 possible cases were solved numerically using mathematical software, namely MATLAB^®^ / Simulink^®^, to obtain 16 sets of vehicle response for subsequent analysis.

**Table 2:** Parameters relevant to quarter vehicle model.

Vehicle parameter	Value or description
Front quarter vehicle sprung mass (kg)	365.4
Rear quarter vehicle sprung mass (kg)	285.1
Front unsprung mass (kg)	43
Rear unsprung mass (kg)	38
Front linear suspension stiffness (Nm^-1^)	24000
Rear linear suspension stiffness (Nm^-1^)	25000
Front suspension stiffness non-linearity	As in Fig 3a
Rear suspension stiffness non-linearity	As in Fig 3a
Front non-linear suspension damping	As in Fig 3b
Rear non-linear suspension damping	As in Fig 3b
Front linearized suspension damping rate (Nsm^-1^)	2126.4
Rear linearized suspension damping rate (Nsm^-1^)	4322.8
Linear tire stiffness (Nm^-1^)	350000
Linear tire damping rate (Nsm^-1^)	1000

## Analysis of simulated vehicle responses

After the completion of computation for all cases, the generated results were compiled for comparative analysis here. Results are in the form of vehicle response in time domain for front and rear quarter vehicle models. As a quarter vehicle model comprises two DOFs only, both sprung and unsprung mass responses are used as the basis for the few comparisons first mentioned in the preceding section. Apart from qualitative evaluation, comparisons are also made quantitatively by considering some derived result parameters. Considering the transient nature of the responses, particularly for sprung mass, several transient response parameters, namely rise time, peak time, maximum fractional overshoot and settling time are considered in the comparisons. In addition, three RMS result parameters commonly used for suspension performance evaluation in a typical study involving quarter vehicle model, namely RMS sprung mass acceleration, RMS dynamic tire load and RMS suspension travel, are also considered. The resulted data is presented in Table 3 for front quarter vehicle model and Table 4 for rear quarter vehicle model.

**Table 3:** Results from all test cases for front quarter vehicle model.

**Result parameter**	**Case 1**		**Case 2**	**Case 3**	**Case 4**	**Case 5**
Rise time (s)	0.1179		0.3101	0.1195	0.0520	0.1390
Peak time (s)	0.3285		0.6470	0.3315	0.2414	0.3166
Maximum fractional overshoot	0.4392		0.2182	0.4382	1.1296	0.2265
Settling time (s)	1.3329		1.5657	1.3365	1.6392	0.9070
RMS sprung mass acceleration (ms^-2^)	2.5303		0.4299	2.1542	7.6792	2.1901
RMS dynamic tire load (N)	1782.4940		170.7891	4855.4763	1853.1432	1937.5973
RMS suspension travel (×10^−3^ m)	13.8585		5.3787	13.5158	22.5059	12.7512
	**Case 6**	**Case 7**	**Case 8**	**Case 9**	**Case 10**	**Case 11**
Rise time (s)	0.3103	0.3101	0.3309	0.0607	0.1484	0.0496
Peak time (s)	0.6473	0.6470	0.6251	0.2593	0.3300	0.2268
Maximum fractional overshoot	0.2181	0.2182	0.1193	0.9531	0.1611	1.1971
Settling time (s)	1.5663	1.5657	0.8920	1.6529	0.6000	1.0662
RMS sprung mass acceleration (ms^-2^)	0.4268	0.4299	0.4058	5.9224	1.7733	8.8947
RMS dynamic tire load (N)	168.0282	170.7891	164.7845	4887.3830	4866.6958	2003.8089
RMS suspension travel (×10^−3^ m)	5.3698	5.3787	3.8354	20.0257	12.0368	23.7880
	**Case 12**	**Case 13**	**Case 14**	**Case 15**		**Case 16**
Rise time (s)	0.3103	0.3309	0.3309	0.0584		0.3309
Peak time (s)	0.6473	0.6257	0.6251	0.2418		0.6257
Maximum fractional overshoot	0.2181	0.1192	0.1193	0.9498		0.1192
Settling time (s)	1.5663	0.8920	0.8920	1.0754		0.8920
RMS sprung mass acceleration (ms^-2^)	0.4268	0.4003	0.4058	7.0037		0.4003
RMS dynamic tire load (N)	168.0282	161.1448	164.7845	4907.1098		161.1448
RMS suspension travel (×10^−3^ m)	5.3698	3.8085	3.8354	19.8887		3.8085

**Table 4:** Results from all test cases for rear quarter vehicle model.

**Result parameter**	**Case 1**		**Case 2**	**Case 3**	**Case 4**	**Case 5**
Rise time (s)	0.0709		0.3497	0.0741	0.0705	0.9859
Peak time (s)	0.2156		0.6085	0.2191	0.2150	-
Maximum fractional overshoot	0.2240		0.1068	0.2246	0.2273	-
Settling time (s)	0.5189		0.8582	0.5216	0.5192	1.7273
RMS sprung mass acceleration (ms^-2^)	4.3804		0.4381	3.9015	4.3834	3.0565
RMS dynamic tire load (N)	1708.0939		140.1934	4868.0161	1707.2397	1931.8855
RMS suspension travel (×10^−3^ m)	8.6453		2.5834	8.3566	8.6556	21.4756
	**Case 6**	**Case 7**	**Case 8**	**Case 9**	**Case 10**	**Case 11**
Rise time (s)	0.3496	0.3497	0.3724	0.0741	0.9624	0.0648
Peak time (s)	0.6093	0.6085	0.5652	0.2191	-	0.1523
Maximum fractional overshoot	0.1070	0.1068	0.0479	0.2246	-	0.1165
Settling time (s)	0.8584	0.8582	0.6232	0.5216	1.7054	0.2370
RMS sprung mass acceleration (ms^-2^)	0.4285	0.4381	0.5549	3.9015	2.1050	10.2966
RMS dynamic tire load (N)	136.8256	140.1934	179.0058	4868.0161	4860.2052	1613.5173
RMS suspension travel (×10^−3^ m)	2.5770	2.5834	1.7851	8.3566	20.2757	7.9740
	**Case 12**	**Case 13**	**Case 14**	**Case 15**		**Case 16**
Rise time (s)	0.3496	0.3728	0.3724	0.5111		0.3728
Peak time (s)	0.6093	0.5666	0.5652	-		0.5666
Maximum fractional overshoot	0.1070	0.0473	0.0479	-		0.0473
Settling time (s)	0.8584	0.6301	0.6232	1.2483		0.6301
RMS sprung mass acceleration (ms^-2^)	0.4285	0.5338	0.5549	6.7419		0.5338
RMS dynamic tire load (N)	136.8256	172.0221	179.0058	4821.5887		172.0221
RMS suspension travel (×10^−3^ m)	2.5770	1.7165	1.7851	10.8010		1.7165

### Evaluation based on individual detail

**Fig 4:**
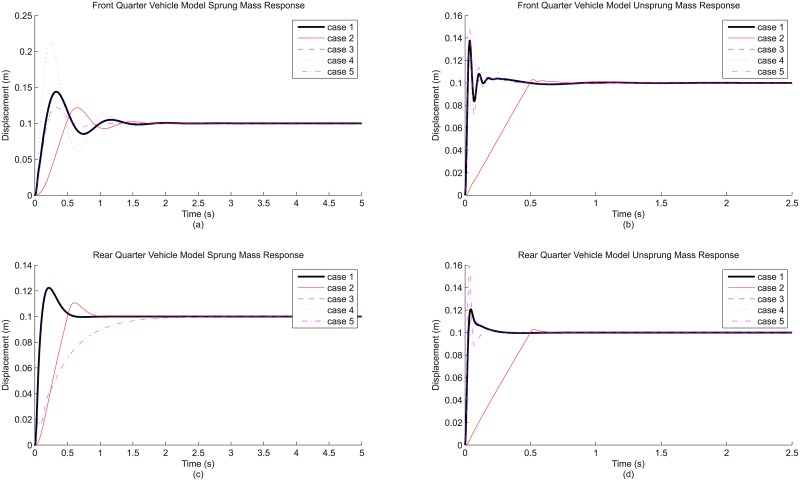
Evaluation on modeling detail addition based on (a) front quarter vehicle model sprung mass response, (b) the corresponding unsprung mass response, (c) rear quarter vehicle model sprung mass response and (d) the corresponding unsprung mass response.

The first comparison involves the cases associated with the addition of individual modeling detail to the base quarter vehicle model, namely case 2 (road input detail), case 3 (tire detail), case 4 (suspension stiffness detail) and case 5 (suspension damping detail). By treating case 1 (base model, no detail added) as the reference, this comparison provides information regarding the effect brought by the inclusion of each of these modeling details. Fig 4 shows the front and rear, sprung and unsprung mass responses for the relevant cases, while the derived differences in result parameters for these cases are shown as Table 5.

**Table 5:** Comparison among relevant cases considering individual modeling details.

Result parameter	Case 1 (reference)	Case 2 difference (%)	Case 3 difference (%)	Case 4 difference (%)	Case 5 difference (%)
*Front quarter vehicle model*					
Rise time (s)	0.1179	163.02	1.36	-55.89	17.90
Peak time (s)	0.3285	96.96	0.91	-26.51	-3.62
Maximum fractional overshoot	0.4392	-50.31	-0.23	157.17	-48.43
Settling time (s)	1.3329	17.47	0.27	22.98	-31.95
RMS sprung mass acceleration (ms^-2^)	2.5303	-83.01	-14.86	203.49	-13.44
RMS dynamic tire load (N)	1782.4940	-90.42	172.40	3.96	8.70
RMS suspension travel (×10^−3^ m)	13.8585	-61.19	-2.47	62.40	-7.99
*Rear quarter vehicle model*					
Rise time (s)	0.0709	393.23	4.51	-0.56	1290.55
Peak time (s)	0.2156	182.24	1.62	-0.28	-
Maximum fractional overshoot	0.2240	-52.31	0.24	1.47	-
Settling time (s)	0.5189	65.39	0.52	0.06	232.88
RMS sprung mass acceleration (ms^-2^)	4.3804	-90.00	-10.93	0.07	-30.22
RMS dynamic tire load (N)	1708.0939	-91.79	185.00	-0.05	13.10
RMS suspension travel (×10^−3^ m)	8.6453	-70.12	-3.34	0.12	148.41

Judging from the responses shown in Fig 4, the effects due to these details are observable. To begin, the addition of road input detail to the base model produces notable changes to the vehicle response (case 2): the rise time is obviously longer, and the maximum overshoot is also lower compared to that of the reference (case 1). This is particularly true for the unsprung mass response. Additionally, from the numerical results in Table 5, all three RMS result parameters are much lower as well. Specifically, the RMS sprung mass acceleration, RMS dynamic tire load and RMS suspension travel are reduced by 90%, 92% and 70% respectively. These changes in vehicle response can logically be explained from the perspective of road input representations used in this study. Step input, although simple, is only a crude representation of a road bump. The unrealistic sudden increase in road displacement translates to unrealistically high impact experienced by both sprung and unsprung masses. On the other hand, ramp input causes significantly less impact which is more realistic, hence the gentler vehicle response and the lower RMS result parameters. Basically, moving from step profile to ramp profile as road input detail changes the responses quite fundamentally.

While it is clear that road input detail has an effect on the simulated vehicle response, the same cannot be said for tire detail. With the inclusion of tire damping alongside tire stiffness, the effect due to tire detail is insignificant, at least for the sprung mass. Referring to the same figure, the sprung mass response (case 3) is almost identical to the reference (case 1). The effect due to tire damping is actually present in the unsprung mass, as can be seen from the huge increase in RMS dynamic tire load (185% in Table 5) which is a parameter indirectly related to unsprung mass response. However, the huge effect is more of a contribution from the use of step road input than the inclusion of tire damping. This is because the vertical tire force, specifically the tire damping force, is extremely large during the initiation of simulation when using step road input as the sudden increase in road displacement causes a very high relative velocity between the road and the unsprung mass. In short, the effect of tire detail is rather inconclusive at this point and has to be observed together with the presence of road detail. This will be elaborated shortly in the next section.

Meanwhile, the effect of having suspension stiffness detail is a mixed one. For instance, the rear sprung mass response (case 4) is almost identical to the reference (case 1), but the front sprung mass response exhibits visible difference, judging from the increased overshoot (Fig 4) and also RMS sprung mass acceleration (203% in Table 5). This is mainly due to the different bump-stop and rebound-stop definitions for front and rear quarter vehicle models, especially the limits of suspension movement. In fact, the effect of having suspension stiffness detail is conditional, since it depends on the combination of suspension movement limit determined by the stops and the extremity of road input which affects the suspension travel. This is very logical, because if the suspension travel does not exceed the limit, then the added detail (definition of the stops) will not change the vehicle response at all. Therefore, it can be said here that suspension stiffness detail does have a noticeable effect, but only when the suspension travel is very large. To be realistic, similar to the case of tire damping, the effect of non-linear suspension stiffness should also be observed with road detail present, although this time it is not a necessity.

Finally, for suspension damping detail, the effect of having non-linear damping is comparable to that achieved by road input detail, as the use of non-linear damping can possibly change the fundamental characteristic of the responses. To illustrate, the rear sprung mass response (case 5) belongs to the over-damping response type, with no overshoot and extremely long rise time which are very different from the reference (case 1). For the front sprung mass, the under-damping response characteristic remains, but the response still exhibits lower overshoot with a much shorter settling time that hints at a much higher damping effect than the reference. The different front and rear response characteristics due to different damping profiles make it difficult to generalize the effect of suspension damping detail to a common trend. However, it can still be said that these observations have good correspondence with the general non-linear damping profile, with very high damping within the initial range of working velocity and much lower damping beyond that (Fig 3b). It is this very high initial damping that gives the highly under-damped, or even over-damped, response for sprung mass. In contrast, the reference has linear damping representation which is constant throughout the entire range of working velocity and produces an averaged damping effect. As a final note, it is also worth mentioning that unlike tire damping and non-linear suspension stiffness, the effect brought by non-linear suspension damping is consistent regardless of the types of road input.

### Evaluation based on selected combined details

As already mentioned, the effects due to the inclusion of tire damping and non-linear suspension stiffness are largely influenced by the types of road input, and they are probably better evaluated also with the much more realistic ramp road input apart from step road input. This calls for the second comparison which is among some selected cases with combined addition of modeling details. Specifically, cases 6 and 7 are put into focus in this comparison as they refer to the situations of having tire detail and suspension stiffness detail respectively, both with road input detail simultaneously. For meaningful comparison, case 2, with only road input detail present, is now the reference. The relevant results are displayed in the form of derived result parameters (Table 6) instead of the original vehicle response due to closeness among the responses. In addition, similar comparison of results from the preceding section (cases 3 and 4 to case 1) are repeated here in Table 6 to aid evaluation.

**Table 6:** Comparison among selected cases considering combined modeling details.

Result parameter	Case 2 (reference)	Case 6 difference (%)	Case 7 difference (%)	Case 1 (reference)	Case 3 difference (%)	Case 4 difference (%)
*Front quarter vehicle model*						
Rise time (s)	0.3101	0.06	0.00	0.1179	1.36	-55.89
Peak time (s)	0.6470	0.05	0.00	0.3285	0.91	-26.51
Maximum fractional overshoot	0.2182	-0.07	0.00	0.4392	-0.23	157.17
Settling time (s)	1.5657	0.04	0.00	1.3329	0.27	22.98
RMS sprung mass acceleration (ms^-2^)	0.4299	-0.73	0.00	2.5303	-14.86	203.49
RMS dynamic tire load (N)	170.7891	-1.62	0.00	1782.4940	172.40	3.96
RMS suspension travel (×10^−3^ m)	5.3787	-0.16	0.00	13.8585	-2.47	62.40
*Rear quarter vehicle model*						
Rise time (s)	0.3497	-0.03	0.00	0.0709	4.51	-0.56
Peak time (s)	0.6085	0.13	0.00	0.2156	1.62	-0.28
Maximum fractional overshoot	0.1068	0.11	0.00	0.2240	0.24	1.47
Settling time (s)	0.8582	0.02	0.00	0.5189	0.52	0.06
RMS sprung mass acceleration (ms^-2^)	0.4381	-2.19	0.00	4.3804	-10.93	0.07
RMS dynamic tire load (N)	140.1934	-2.40	0.00	1708.0939	185.00	-0.05
RMS suspension travel (×10^−3^ m)	2.5834	-0.25	0.00	8.6453	-3.34	0.12

The result parameter values in Table 6 indicate that for both cases 6 and 7, the vehicle responses are actually near identical to the reference (case 2). This is applicable to front and rear, sprung and unsprung mass responses. Here, it can be seen that the effect of having tire damping is actually small, with less than one percentage difference for most result parameters (case 6). The RMS dynamic tire load remains the result parameter with the largest difference, but the difference is significantly smaller compared to the huge percentage difference in the previous comparison. Considering also that tire damping is generally much less than suspension damping, the usual practice of ignoring tire damping in quarter vehicle modeling seems acceptable as long as the road representation is sufficiently close to the reality. For non-linear suspension stiffness, the differences for all result parameters are even reduced to zero percentage (case 7), technically implying that the responses are identical to the reference (case 2). The same justification as described in previous comparison applies: with the realistic ramp input, the resulted suspension travel also becomes realistic and smaller; in the simulation, the small suspension travel never exceeds the limit, giving no effect at all. In other words, although in the previous comparison the effect due to non-linear suspension stiffness is noticeable, in reality this detail does not affect the response much.

### Significance of each modeling detail

**Fig 5:**
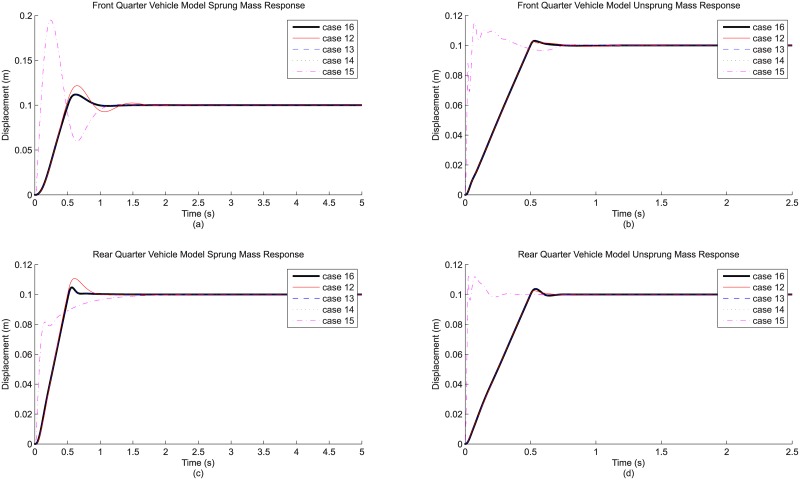
Evaluation on modeling detail reduction based on (a) front quarter vehicle model sprung mass response, (b) the corresponding unsprung mass response, (c) rear quarter vehicle model sprung mass response and (d) the corresponding unsprung mass response.

So far, the comparisons carried out have proven that the various modeling details do have effects on the response of a quarter vehicle model, although their magnitudes vary. However, strictly speaking, the analysis up to this point is limited to the quantification of the effect, and little is known regarding the portion of model accuracy contributed by these details. In this section, a comparison that is complementary to the previous ones is made in an attempt to determine the contribution of accuracy made by each of the four modeling details. This is carried out from a different perspective by using reverse deduction, that is, how much the response deviates from reference (most detailed representation) when each detail is taken away, obviously with the core assumption that the closer a model is to the physical representation, the more accurate the response will be. Consequently, this comparison involves cases 12, 13, 14 and 15 as they can be reversely interpreted as having the suspension damping detail, suspension stiffness detail, tire detail and road input detail omitted from the fully-featured model in case 16 which serves as the reference here. The vehicle responses for the said cases are shown in Fig 5, while the accuracy contributions are deduced from the differences in result parameter values stated in Table 7.

**Table 7:** Comparison among relevant cases for evaluation on accuracy contribution.

Result parameter	Case 16 (reference)	Case 12 difference (%)	Case 13 difference (%)	Case 14 difference (%)	Case 15 difference (%)
*Front quarter vehicle model*					
Rise time (s)	0.3309	-6.23	0.00	0.00	-82.35
Peak time (s)	0.6257	3.45	0.00	-0.10	-61.36
Maximum fractional overshoot	0.1192	82.94	0.00	0.04	696.64
Settling time (s)	0.8920	75.59	0.00	0.00	20.56
RMS sprung mass acceleration (ms^-2^)	0.4003	6.60	0.00	1.36	1649.51
RMS dynamic tire load (N)	161.1448	4.27	0.00	2.26	2945.16
RMS suspension travel (×10^−3^ m)	3.8085	41.00	0.00	0.71	422.22
*Rear quarter vehicle model*					
Rise time (s)	0.3728	-6.22	0.00	-0.11	37.10
Peak time (s)	0.5666	7.54	0.00	-0.25	-
Maximum fractional overshoot	0.0473	126.19	0.00	1.29	-
Settling time (s)	0.6301	36.23	0.00	-1.10	98.11
RMS sprung mass acceleration (ms^-2^)	0.5338	-19.71	0.00	3.97	1163.09
RMS dynamic tire load (N)	172.0221	-20.46	0.00	4.06	2702.89
RMS suspension travel (×10^−3^ m)	1.7165	50.13	0.00	4.00	529.25

Based on the percentage differences of the result parameters, it is very clear that road input detail contributes most to the accuracy of the sprung and unsprung mass responses. In qualitative terms, with the removal of road input detail, the unsprung mass response is much sharper due to the harsh step road input, while the sprung mass response can have different characteristics depending on the front and rear non-linear damping profiles. Overall, the change in responses (case 15) relative to the reference (case 16) is of similar magnitude to the corresponding observation in earlier section ‘Evaluation based on individual detail’, so the finding here is basically consistent to the information from previous comparisons. By switching between the basic step road input and the refined ramp road input, the change in vehicle response remains fundamental regardless of the influences from other modeling details.

The second largest model accuracy is contributed by the suspension damping detail, judging from the overall solid percentage differences stated in Table 7. Consistently, as seen from Fig 5, for unsprung mass response (case 12), the variation from the reference (case 16) exists, but is not very prominent. For sprung mass response, however, the variation is visible. Furthermore, in this comparison, a general trend can be observed: with the removal of suspension damping detail, there is an effect of lower damping based on greater response overshoot and longer settling time. This is reversely in agreement to the relevant observation in earlier section ‘Evaluation based on individual detail’ and can be similarly attributed to the difference between non-linear and linear damping characteristics discussed earlier. However, relative to the first comparison (Fig 4 as well as Table 5), the effect due to suspension damping detail becomes smaller here. This makes suspension damping detail less significant than the road input detail.

Meanwhile, both tire detail and suspension stiffness detail seem to contribute very little to the accuracy of vehicle response. For example, the omission of tire damping records only single-digit percentage differences for all evaluated result parameters (Table 7). Graphically, the responses (case 14), especially sprung mass response, almost coincide with the reference (case 16). This is consistent to the previous comparisons in earlier sections ‘Evaluation based on individual detail’ and ‘Evaluation based on selected combined details’. Based on all the observations, the most that can be said regarding tire detail is that this detail only shows observable effect on the unsprung mass response, particularly in the form of increased RMS dynamic tire load, but even so it is still only by single-digit percentage. Thus, the contribution due to tire damping as the tire detail is very limited. Finally, the suspension stiffness detail (case 13) does not even contribute anything compared to the reference (case 16) judging from the zero percentage difference across all result parameters. The same justification as in preceding section ‘Evaluation based on selected combined details’ applies. So, for a fully-featured quarter vehicle model, unless the extremity of road input is very high, the modeling of bump-stop and rebound-stop for non-linear stiffness representation does not play a role in determining the overall response accuracy.

At this point it is worth to make an overall evaluation. The importance or significance of a modeling detail is basically constituted by the magnitude of the effect brought by the detail as well as the accuracy contribution from it. Inferring from the outcomes of various comparisons up to this point, it can be said that road input is the most significant detail to a quarter vehicle model, as its presence changes the vehicle response very fundamentally and makes the greatest difference in terms of accuracy. Road input aside, suspension damping detail is also of similar significance as the detailed non-linear damping representation is capable of giving vastly different response characteristics, and this is both notable and consistent. Conversely, on the other side of thing, suspension stiffness detail and tire detail are much less significant when quarter vehicle model is concerned. The former’s presence, although capable of making a difference to the response, is only felt conditionally. More often than not, it gives no contribution to accuracy. Lastly, the latter, in the form of the addition of tire damping, is also very insignificant to quarter vehicle modeling as its presence has little effect and accuracy contribution, especially for sprung mass response which is usually the focus for ride-related studies employing this model. Together, these inferences give knowledge on significance to allow more informed decision to be made between the inclusion and exclusion of a particular detail in quarter vehicle modeling.

### Further assessments and discussions

In this section, additional evaluations are carried out regarding possible incremental modeling detail addition, as well as possible further refinements to the modeling details already introduced. For the former, the suspension damping detail is put into focus because it is acknowledged that there are many intermediate states between the linear and fully non-linear damping that are also commonly adopted, including the linear but asymmetrical damping (also called bi-linear damping) and the more involved piece-wise function modeling approach which are definitely worth being evaluated.

**Fig 6:**
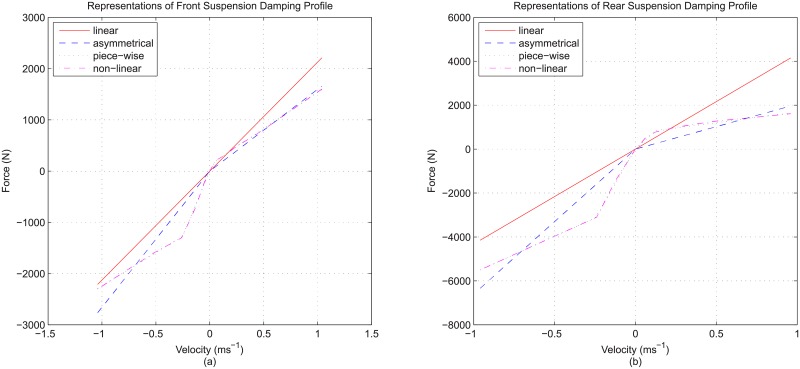
Various representations of (a) front damping profile and (b) rear damping profile used in this study.

For this further evaluation, the comparison is made among several suspension damping representations with increasing refinement. In total, four representations are considered: while the basic linear damping and the fully non-linear damping considering lookup table approach have already been introduced, two other damping representations, namely linear but asymmetrical damping and the two-piece, piece-wise damping modeling are considered here. These are the intermediate states that sit between linear and non-linear damping. For asymmetrical damping, the damping effect can still be seen as linear, but it is a step forward with different damping rates for the jounce and rebound directions. Meanwhile, the piece-wise damping is another step further by approximating the non-linearity of damping profile using two-piece mathematical function for each direction, giving a semi-linear representation closer to the actual non-linearity. To clarify further, these two damping representations, together with the linear and non-linear damping already known, are illustrated in Fig 6.

**Fig 7:**
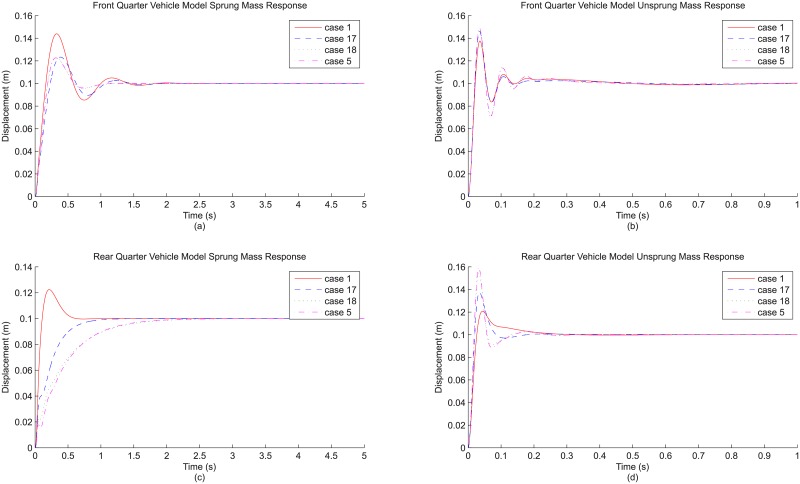
Comparison among incremental damping details based on (a) front quarter vehicle model sprung mass response, (b) the corresponding unsprung mass response, (c) rear quarter vehicle model sprung mass response and (d) the corresponding unsprung mass response.

In this comparison, the two newly introduced damping representations are seen as incremental details and are referred to as cases 17 and 18 which is simply a continuation from the existing nomenclature. Case 17 refers to the addition of asymmetrical representation as the suspension damping detail while case 18 refers to the use of piece-wise modeling as the detail. Consistent to the incremental nature, the comparison is done in a sequential manner, following the order of no damping detail or linear damping (case 1), with asymmetrical damping as detail (case 17), with piece-wise damping representation (case 18) and finally with fully non-linear damping using lookup table representation as the detail (case 5). Because each is a step superior to the immediate previous one, the accuracy improvement can be determined for each successive incremental detail by evaluating the vehicle responses and the relative percentage differences which take the immediate previous case as the reference. The relevant results are shown in Fig 7 and Table 8 respectively.

**Table 8:** Relative differences among cases with incremental damping details.

Result parameter	Case 1 (first reference)	Case 17 difference (%) relative to case 1	Case 18 difference (%) relative to case 17	Case 5 difference (%) relative to case 18
*Front quarter vehicle model*				
Rise time (s)	0.1179	45.38	-18.67	-0.29
Peak time (s)	0.3285	17.99	-18.01	-0.38
Maximum fractional overshoot	0.4392	-46.87	-1.81	-1.15
Settling time (s)	1.3329	-1.37	-29.67	-1.90
RMS sprung mass acceleration (ms^-2^)	2.5303	-8.78	-5.95	0.89
RMS dynamic tire load (N)	1782.4940	3.18	6.20	-0.80
RMS suspension travel (×10^−3^ m)	13.8585	-2.81	-4.70	-0.67
*Rear quarter vehicle model*				
Rise time (s)	0.0709	582.23	108.08	-2.05
Peak time (s)	0.2156	-	-	-
Maximum fractional overshoot	0.2240	-	-	-
Settling time (s)	0.5189	63.52	113.42	-4.62
RMS sprung mass acceleration (ms^-2^)	4.3804	-17.80	-14.85	-0.32
RMS dynamic tire load (N)	1708.0939	1.88	7.41	3.36
RMS suspension travel (×10^−3^ m)	8.6453	72.45	34.76	6.89

As expected, the sprung mass response changes with every incremental damping detail. Basically, it goes towards the direction of higher damping effect with every incremental step, and this is consistently observable for both front and rear responses. Correspondingly, because a quarter vehicle model has only two DOFs, the other mass, namely the unsprung mass, has response that is more oscillatory with the same successive damping detail increment. The justification for these observations is again the same as that described in previous comparisons. The basic linear damping (case 1) is linearized from the original damping profile and has a constant damping rate which is overall less as it averages the non-linearity. The asymmetrical damping (case 17) introduces slightly lower damping rate in jounce direction but notably higher damping rate in rebound direction, which is a more correct representation. For both piece-wise (case 18) and fully non-linear (case 5) representations, because of the division of each direction into two sections with the initial part having much higher damping, they are obviously the most correct representations in this comparison. Therefore, it is not surprising that in a general way, each increment in detail is loosely associated with higher damping effect. The more interesting observation here, however, is how close the vehicle responses are between cases 18 and 5. From Fig 7, the responses for both sprung and unsprung masses are close to overlapping, and from Table 8 the relative percentage differences for all result parameters are of single digit, especially for the front quarter vehicle model which records mostly less than one percentage difference when stepping up from piece-wise representation to fully non-linear representation. This implies that the piece-wise function modeling approach is already capable of providing accurate representation of damping in a quarter vehicle model simulation. Considering also that this representation is simpler to model than the fully non-linear damping using lookup table, it seems to strike a better balance between accuracy and simplicity concerning quarter vehicle modeling.

**Fig 8:**
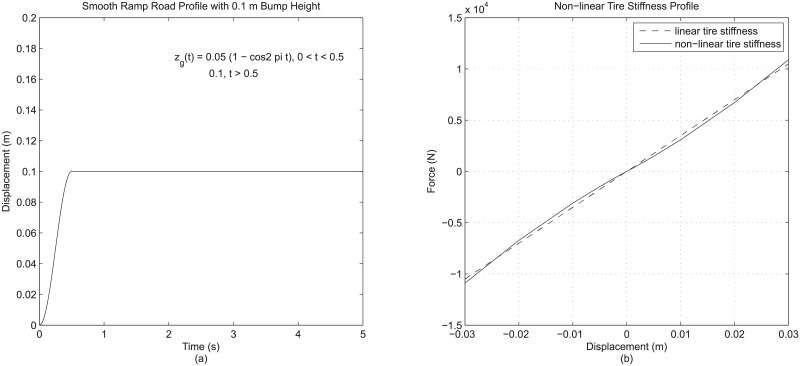
(a) Smooth ramp road profile and (b) non-linear tire stiffness profile used in further evaluation.

Meanwhile, the second evaluation in this section evaluates the possibility of further refinements to the existing modeling details. Of the four modeling details that are present in the fully-featured quarter vehicle model in case 16, the suspension stiffness and suspension damping details, which involve the fully non-linear representations, are basically very well-defined. In contrast, for road input detail and tire detail, there is still some room for further refinement. For road input detail, within the context of transient event involving a vehicle hitting a bump, it is possible to have a closer-to-reality representation by adopting a smooth ramp profile based on sinusoidal definition, as in Fig 8a, instead of the basic ramp profile as used previously. Meanwhile, for tire detail, although the combination of linear tire stiffness and tire damping is typically seen as a comprehensive vertical tire model in the context of vehicle simulation, it is still possible to achieve higher fidelity through non-linear load curve that acts as non-linear tire stiffness representation, as shown in Fig 8b. These two refinements are incorporated in the fully-featured model (as in case 16), and the resulted models are designated as case 19 and case 20. For case 19, the smooth ramp profile is adopted as the road input detail in place of the basic ramp profile; for case 20, the non-linear tire stiffness is adopted alongside tire damping to form the tire detail. Table 9 shows the comparison of responses due to these further detail refinements to the original fully-featured model (case 16).

**Table 9:** Comparison among selected cases involving further modeling detail refinements.

Result parameter	Case 16 (reference)	Case 19 difference (%)	Case 20 difference (%)
*Front quarter vehicle model*			
Rise time (s)	0.3309	-23.27	-0.51
Peak time (s)	0.6257	-6.28	-0.16
Maximum fractional overshoot	0.1192	1.23	3.90
Settling time (s)	0.8920	-4.32	-0.38
RMS sprung mass acceleration (ms^-2^)	0.4003	23.85	0.25
RMS dynamic tire load (N)	161.1448	21.70	-0.16
RMS suspension travel (×10^−3^ m)	3.8085	20.41	0.36
*Rear quarter vehicle model*			
Rise time (s)	0.3728	-27.74	-0.54
Peak time (s)	0.5666	-7.06	0.67
Maximum fractional overshoot	0.0473	-22.22	11.87
Settling time (s)	0.6301	-5.55	1.52
RMS sprung mass acceleration (ms^-2^)	0.5338	-9.72	-0.42
RMS dynamic tire load (N)	172.0221	-10.12	-0.54
RMS suspension travel (×10^−3^ m)	1.7165	3.74	-0.96

Judging from the results in Table 9, the adoption of these two possible further refinements to the fully-featured quarter vehicle model give some further changes to vehicle response. The smooth ramp profile (case 19), although differs only slightly from the basic ramp profile, still results in quite a notable effect on the vehicle response based on the rather solid percentage differences for most of the derived result parameters. This again confirms the significance of road input detail in a quarter vehicle model and the importance of having a fine representation of road input detail for improved model accuracy. Meanwhile, it is observed that the adoption of non-linear tire stiffness (case 20) affects, in particular, the maximum overshoot of the sprung mass response, though not very significantly. This is unlike the incorporation of tire damping in the earlier analysis which is found to affect mainly the RMS dynamic tire load. Consequently, it can be said that different aspects of tire detail in a quarter vehicle model affects different aspects of vehicle response.

**Fig 9:**
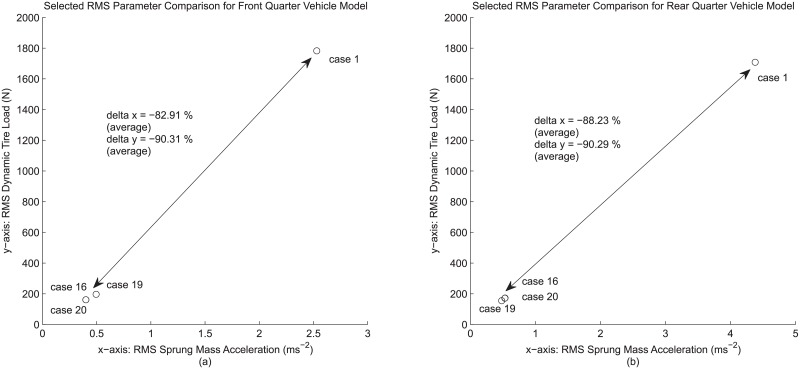
Selected RMS parameter comparison between fully-featured models and the base model, concerning (a) front quarter vehicle model and (b) rear quarter vehicle model used in this study.

Lastly, both cases 19 and 20, together with case 16, are compared against case 1 for an overall evaluation between these group of fully-featured quarter vehicle models (in which all four modeling details are present) and the basic, linear quarter vehicle model. Fig 9 shows such comparison involving two RMS parameters that are commonly used for suspension performance assessment, namely the RMS sprung mass acceleration (ride performance indicator) and RMS dynamic tire load (road holding performance indicator). The comparison shows that the models in cases 16, 19 and 20 are a huge step from that in case 1 with an average of 80% to 90% difference for both RMS parameters. Considering that higher fidelity in modeling results in greater model accuracy, it can be said that a fully-featured quarter vehicle model with all the possible details present is a significant improvement over the typically adopted basic quarter vehicle model.

In general, improvement in model accuracy concerning vehicle simulation is desirable because it is expected that such model enhancement will eventually lead to superior vehicle suspension performance. In the design and development of controllable vehicle suspension, such as an active suspension system [26], the less model uncertainty basically allows better controller parameters to be obtained. It follows that improved performance can be obtained due to an accurate model leading to appropriate selection of control gains [26]. Similarly, the end result of having better, or more optimized, parameters is also applicable to the common passive vehicle suspension. This is because lumped-mass vehicle models can be used in initial optimization to outline a region of interest for suspension parameters prior to final determination of optimum parameters [22, 23], and a more accurate model gives rise to a narrower or more definite region of interest which improves the possibility of finding the global optimum suspension parameters. This is also true for non-conventional passive vehicle suspensions like the suspension with inerter, in which model enhancement such as the inclusion of suspension damping detail (non-linear damping with much higher damping in the initial region) ensures truly optimum suspension performance with the selection of truly optimized inerter parameter, rather than an apparently optimized parameter by considering only linearized damping (with an averaged, lower damping effect which deviates from actual characteristic).

As a final thought, while it is demonstrated that the inclusion of modeling details leads to improved accuracy in the predicted vehicle response, it is worth to discuss the difference between this and similar accuracy improvement brought by the use of vehicle models with greater DOFs. Basically, the inclusion of various vehicle modeling details increases the fidelity of the model within the same DOFs and brings the model closer to the physical representation. Meanwhile, adopting a vehicle model with greater number of DOFs opens the possibility for more features of a vehicle to be incorporated, which potentially improves the accuracy. These are two different approaches to more accurate vehicle modeling. Therefore, although the inclusion of modeling details does bring improved accuracy, additional DOFs, such as the roll, pitch, bending and torsional motions of the vehicle body, need to be incorporated when vehicle responses related to these motions are concerned. On the other hand, for purely vertical vehicle dynamic analysis or vehicle ride analysis, the use of a fully-featured quarter vehicle model with all the possible modeling details added means that meaningful quantitative vehicle analysis with a simple model becomes viable now.

## Conclusion

To sum up, the outcomes of this study have shown that various modeling details associated with a quarter vehicle model simulation do have effect on vehicle response, albeit to different extents. In particular, road detail changes the response fundamentally, damping detail affects the response significantly and consistently, stiffness detail similarly affects the response albeit only conditionally, while tire detail has rather insignificant effect. Interestingly, for non-linear damping modeling, the vehicle response due to piece-wise approximation approach is sufficiently close to fully non-linear representation approach, to the point that the former actually demonstrates a better compromise between significance and modeling convenience. Overall, a fully-enhanced quarter vehicle model with all the details added is a significant improvement compared to the base model. Looking at these outcomes, it is certain that the commonly adopted basic quarter vehicle model still has a lot of untapped potentials. In fact, a better trade-off between model simplicity and model accuracy can be readily achieved through the inclusion of the significant modeling details. Although this study does not reveal anything about the absolute or global accuracy of quarter vehicle model, it does convey an important message: that it is not all about the number of DOFs when it comes to vehicle modeling; it is also about the non-DOF-related details that are equally significant. By quantifying their significance, this study equips researchers the knowledge to decide the inclusion or exclusion of a modeling detail when using quarter vehicle model in their related work.

## Supporting information

S1 Table Result parameters for all cases. (XLSX)

S2 Table All responses for front quarter vehicle model. (XLSX)

S3 Table All responses for rear quarter vehicle model. (XLSX)
